# Oral Health-Related Knowledge, Attitude and Practice among Patients in Rural Areas around Cluj-Napoca, Romania

**DOI:** 10.3390/ijerph19116887

**Published:** 2022-06-04

**Authors:** Radu Marcel Chisnoiu, Ada Gabriela Delean, Alexandrina Muntean, Doina Iulia Rotaru, Andrea Maria Chisnoiu, Sanda Ileana Cimpean

**Affiliations:** 1 Department of Odontology, Endodontics and Oral Pathology, “Iuliu Hațieganu” University of Medicine and Pharmacy, 33 Motilor Street, 400001 Cluj-Napoca, Romania; marcel.chisnoiu@umfcluj.ro (R.M.C.); ada.delean@umfcluj.ro (A.G.D.); doina.rotaru@umfcluj.ro (D.I.R.); sanda.cimpean@umfcluj.ro (S.I.C.); 2 Department of Pedodontics, “Iuliu Hațieganu” University of Medicine and Pharmacy, 31 Avram Iancu Street, 400083 Cluj-Napoca, Romania; alexandrina.muntean@umfcluj.ro; 3 Department of Prosthodontics, “Iuliu Hațieganu” University of Medicine and Pharmacy, 32 Clinicilor Street, 400006 Cluj-Napoca, Romania

**Keywords:** oral health, self-perception, knowledge, attitude, practices

## Abstract

The intent of the KAP (knowledge, attitude, practices) survey is to collect information about a specific target group related to their knowledge, what they consider to be known, and what they do about a particular topic. The aim of this study was to identify the level of importance of oral health, especially the level of knowledge, attitude and practices of rural patients around Cluj-Napoca, Romania. Material and methods: A questionnaire consisting of five parts was used, trying to assess the self-perception of oral health, knowledge and attitude towards oral health, dental hygiene practices and eating habits. A clinical examination was also performed in order to identify the number of missing teeth, the caries and the presence of calculus. Results: The study revealed that most patients have an average and good self-perception of oral health, although three-quarters of them have calculus deposits, and about half of them have more than three carious lesions and at least one extracted tooth. There is also a discrepancy between declared knowledge of oral health, eating habits and dental practices. Conclusions: The basic knowledge of rural patients about oral health can be considered satisfactory, but the practices, unfortunately, do not reflect this knowledge.

## 1. Introduction

Many factors are relevant when considering oral health. Tobacco, alcohol, oral hygiene, nutrition and even general eating habits influence the development of caries and periodontal diseases. There have been signs of improvement in oral health in industrialized countries, where prevention programs exist and operate, and efforts are being made to regulate and adjust sugar consumption, fluoride use and lifestyle [1,2]. There is evidence that proper oral health education leads to better oral care habits. At the same time, a positive attitude regarding oral health practices encourages the improvement of oral health habits. In order to create a relevant and appropriate program for health education, it is essential to assess the level of knowledge, attitude and practice of the population regarding oral health [3,4].

The diseases of the oral cavity have a significant impact on the social life of the individual. Periodontal disease can lead to tooth loss, which, in turn, affects the individual physically, emotionally and even financially [5,6].

The pathology of the oral cavity often affects the aesthetics as well as the functionality of the dento-maxillary apparatus. The mentioned factors play an important role in the daily life of the individual and their social relations, which, in turn, will affect the quality of life. With a good health education program, knowledge of oral diseases and ways to combat them, the chances of minimizing the effects of oral diseases will increase significantly [7].

The most relevant diseases of the oral cavity are dental caries and periodontal disease. The goal should be to raise awareness of the causes of these diseases and to inform patients about how to reduce and even eliminate them. The most important target group for information on preventive measures are the parents, teachers and medical staff. It was noted that a higher level of information is associated with improved hygiene and, also, with a more positive attitude towards oral health [7].

Consistent with this, a study conducted in Sweden stated that socio-economic factors and the origin of the individuals surveyed are determinants of oral health habits. The results also showed that regular dental check-up is associated with better oral health, and if this behavior is established in childhood, it will persist throughout the whole life [8].

The KAP survey (knowledge, attitude, practices) is based on questions formulated in standardized questionnaires that can provide quantitative and qualitative information. The intent of such a study is to collect information about a specific target group related to their knowledge, what they consider to be known, and what they do about a particular topic (in our case, oral health).

The aim of this study was to identify the level of importance for oral health, especially the level of knowledge, attitude and practices of rural patients (living in rural areas or countryside, outside towns and cities) who attend the specialized service within the Odontology Discipline of the Faculty of Dental Medicine, “Iuliu Hațieganu” University of Medicine and Pharmacy Cluj-Napoca, located in northwest Romania.

## 2. Materials and Methods

The study was conducted between 2020 and 2021, including a group of 258 patients (138 men, 120 women) aged between 16 and 69 years. The group followed the guidelines of the World Health Organization’s oral health study guide, considering that, at this age, all teeth except the third molars were erupted. The level of oral health knowledge (OHK), oral health attitude (OHA), oral health practice (OHP) and self-perception of patients’ oral health were assessed.

A questionnaire, which took a maximum of 15 min to complete, was used.

In order to better design the questionnaire and better understand what type of survey is best, the WHO Study Guide and the WHO Knowledge, Attitude and Practice Survey Development Guide [4,9] were consulted. Questionnaires used in other studies [2,3,7,10,11] were also analyzed, and then an original questionnaire was formulated.

It consisted of 17 question-and-answer items—simple questions related to respondents’ knowledge, attitudes and practices regarding oral hygiene. Questions related to oral health referred to the causes of dental caries, the reason for oral hygiene, what fluoride is, etc. Questions were also asked about teeth brushing, as well as questions about toothache and oral health information.

The questionnaire consisted of 5 parts:

1. Questions related to self-perception of oral health
2. Knowledge of oral health
3. Attitude towards oral health
4. Dental hygiene practices
5. Eating habits

No exclusion criteria were applied, excepting cognitive disorders inhibiting the response to a questionnaire.

The clinical examination was performed by a single investigator, and as instruments, the examination kit and the air spray were used. It was aimed to identify the number of missing teeth, the number of caries and the presence of calculus in the lower incisor group. The DMFT (decayed, missing, filled teeth) index was not used, but rather a simplified recording method due to the limited time (maximum 10 min).

Criteria used:

1. For caries recording:
  - To be present, regardless of the number of affected areas;
  - Root rests were recorded as caries;
  - Third molars were excluded.
2. For dental calculus recording:
  - Only the presence of supragingival calculus covering more than one-third of the lingual surface of the lower incisors was taken into account;
  - Missing teeth recording;
  - Third molars were excepted;
  - Root rests were not taken into account.

The first three patients were examined twice, once a week, to make sure that the variations in the examination were minimal.

The aim of this study was explained to all participants, and each agreed to participate, understanding that their identity data are not used and that they have the right to refuse their contribution. This study was approved by the Ethics Committee of our University (approval 160/2018).

### Analysis and Statistics

Statistical analysis and descriptive statistics were determined using Microsoft Excel (Microsoft Corp., Redmond, WA) and SPSS (Statistical Package for Social Sciences software 22.0, Chicago, IL, USA). The data obtained were saved in an Excel file, and the statistical analysis took into account the patients’ age, sex, clinical data and questionnaire results. Where *p* was calculated, the values lower than 0.05 were considered statistically significant.

A reliability test was used to test the stability and consistency of the questionnaire. The Cronbach’s alpha test was used, and a value of 0.738 was obtained, indicating high internal reliability.

## 3. Results

Considering the age and sex of the patients, although there were differences between the answers given by the participants in the questionnaire, they were not statistically validated (*p* > 0.05).

Regarding the patient’s perception about their own oral health, it can be stated that most have a medium or good opinion about it, and only a small part, especially females, consider that they have poor oral health. (Table 1).

Regarding the knowledge that patients have about oral health, most said that they had obtained this information since childhood from the family, the next important source being the dentist. It should be noted that the vast majority of patients are aware that brushing their teeth, reducing their consumption of sweets and regular visits to the dentist result in adequate oral health (Table 2).

Regarding the attitude, the vast majority of patients consider oral health very important; however, the high cost of dental treatments and the fear of them are the most important reasons to avoid visiting the dentist. However, almost one-quarter of respondents said they did not avoid these visits. Another interesting result was that in three-quarters of the cases, the pain was the reason for visiting the dental office (Table 3).

Regarding patients’ practices, almost half of the patients declare that they brush twice a day and use mouthwash as an additional oral cleansing aid (Table 4).

Patients’ eating habits involve the daily consumption of sweetened drinks or food for about half of the respondents (Table 5).

The clinical examination revealed that three-quarters of the examined patients had calculus and at least one carious lesion or an extraction.

## 4. Discussion

The World Health Organization has conducted numerous studies to monitor the progress of dental caries, especially in children. The first global map of dental caries prevalence was made in 1969 and showed that the prevalence of tooth decay was very high in industrialized countries and generally low in developing countries [12]. The elaboration of databases and the increase in the number and types of epidemiological studies allowed the assessment of the evolution pattern of dental caries [13,14]. Thus, the latest studies show a decline in dental caries in industrialized countries and an increase in developing countries [12].

The decline of dental caries in developed countries is the result of public dental health measures, accompanied by changes in living standards and lifestyle [15]. It should be noted that despite the improvements, dental caries, as a disease, is not eradicated but kept under control to some extent [12].

KAP surveys can identify gaps in knowledge, cultural beliefs or patterns of behavior. They can identify why people have certain health behaviors and the factors that influence their attitudes. KAP surveys can also assess important methods of communication and sources for the prevention of oral diseases.

Studies have shown that a sound knowledge of oral health leads to better oral care practices [3,16]. Therefore, it is important to improve both the knowledge and habits of patients.

In the present study, it was found that the most common source of information about oral health was the family. In other studies [17,18], the most common source of information was stated to be the media (TV, radio). However, this information is often inaccurate. Therefore, a more organized and better-informed framework should be considered. A viable option is represented by schools and by training teachers in explaining correct practices. The teacher is a key player in communication and has the opportunity to train young people. The oral health of children and adolescents could be improved by corrected oral hygiene practices.

Furthermore, considering the relationship between parents and children, it is important to educate parents so that they can give adequate advice to their children [19]. At the same time, a recent Swedish study stated that children could also influence their parents’ behavior, attitude and knowledge [20].

Although the vast majority of patients say they have knowledge of oral health (what it means and how to maintain it), including the use of fluoride in caries prevention, most acknowledge that the reason for visiting the dentist is pain or discomfort caused by dental problems; the main reason they invoke for avoiding the dentist’s office is the high costs. These results contradict the findings of the studies of Parveen et al. [21] and Nagarajappa et al. [22] but are consistent with data reported by Sen et al. [17], which indicates that oral health concerns differ from one region to another and cannot be generalized.

The fact that women brush their teeth more often is consistent with the results obtained in other studies [23,24].

In general, according to the results obtained in this study, few patients use dental floss (approx. 28%). Many do not even know about this additional oral care aid. Some studies [17,25] have reported much lower percentages of patients who use floss, but there are also studies that have reported a percentage of up to 44% of patients who use it [26]. However, the general opinion is that dental floss used as an addition to interdental hygiene has not been sufficiently promoted in dental education [27]. However, tooth showers are unknown to all patients. Perhaps all these additional oral care aids should be part of the basic arsenal of oral hygiene.

As the present study shows, there is a discrepancy between knowledge of oral health, on the one hand, and eating habits and dental practices, on the other. Although the vast majority of respondents (97%) consider that the consumption of sweets affects oral health, about 50% stated that they usually eat sweetened food or drink daily. Furthermore, the majority of respondents (approx. 90%) believe that brushing their teeth prevents caries as well as gingival problems; nevertheless, approx. 43% brush their teeth at most once a day. These results are in line with those reported by P. Scaglia and N. Sen [17,28] and can be attributed to the emotions of patients and their desire to show their knowledge of dental hygiene, but when they need to declare what their personal habits are, they sincerely declare their daily habits. Patients’ knowledge of oral health is extremely important, but unfortunately, if not put into practice, oral health status can hardly be improved [29].

An interesting observation in our study is that most patients have an average and good self-perception of oral health, although three-quarters of them have calculus deposits, and about half of them have more than three carious lesions and at least an extracted tooth. Consistent with the results of other studies [13,15], most carious lesions were detected on clinical examination, as patients were unaware of their existence. It is difficult to say why the incidence is high, but it is most likely due to the high cost of dental services and poor oral hygiene. The positive perception is consistent with that described by Jokovic and Locker [30] and can be explained by the fact that as long as there is nothing visible or objective, such as bleeding, pain or mobility, patients consider oral health status to be appropriate.

This study certainly has some limitations. It should be considered that the study group was not very large, but, also, this study can be considered a pilot one, especially since there are no data on the status of oral health in this region. Moreover, the clinical environment in which patients responded to the questionnaire may have put pressure on them and made them overestimate their answers regarding knowledge, oral health habits and visits to the dentist and underestimate their answers regarding eating habits.

## 5. Conclusions

The basic knowledge of rural patients about oral health can be considered satisfactory, but the practices, unfortunately, do not reflect this knowledge. Moreover, the additional oral care aids are, for many patients, unknown. At the same time, the collected clinical data reveal that the dental practices of the patients are not in accordance with the declared knowledge. This study shows that there is a need to educate patients about oral health issues and oral health maintenance methods.

Despite the limitations of this study and considering that, to the knowledge of the authors, there is no such study at the regional level, it fills a gap in epidemiological information that may provide a basis for further research or even be useful in planning dental prevention in this area.

## Figures and Tables

**Table 1:** Patients’ self-perceptions of oral health status.

Parameter	Evaluation Index	Total [*n* (%)]	Men [*n* (%)]	Women [*n* (%)]
Gum and teeth health	Excellent	30 (11.6)	18 (13)	12 (10)
	Very good	36 (13.9)	18 (13)	18 (15)
	Good	60 (23.2)	36 (26)	24 (20)
	Medium	84 (32.5)	42 (30.4)	42 (35)
	Bad	24 (9.3)	12 (8.6)	12 (10)
	Very bad	18 (6.9)	6 (4.3)	12 (10)
	Do not know	6 (2.3)	6 (4.3)	0
Teeth appearance	Very good	60 (23.2)	30 (21.7)	30 (25)
	Satisfactorily	78 (30.2)	48 (34.7)	30 (25)
	Less satisfactorily	54 (20.9)	30 (21.7)	24 (20)
	Unsatisfactorily	66 (25.5)	30 (21.7)	36 (30)

**Table 2:** Oral health knowledge.

Parameter	Evaluation Index	Total [*n* (%)]	Men [*n* (%)]	Women [*n* (%)]
Information source	Friends, family	108 (41.8)	48 (34.7)	60 (50)
	School	36 (13.9)	18 (13)	18 (15)
	TV/radio	48 (18.6)	30 (21.7)	18 (15)
	Dentist	66 (25.5)	42 (30.4)	24 (20)
Tooth brushing prevents caries	Agree	246 (95.3)	126 (91.3)	120 (100)
	Disagree	6 (2.3)	6 (4.3)	0
	Do not know	6 (2.3)	6 (4.3)	0
Tooth brushing prevents gingival bleeding and tooth loss	Agree	228 (88.3)	120 (86.9)	108 (90)
	Disagree	12 (4.6)	12 (8.6)	0
	Do not know	18 (6.9)	6 (4.3)	12 (10)
Consumption of sweets increases the caries risk	Agree	252 (97.6)	132 (95.6)	120 (100)
	Disagree	6 (2.3)	6 (4.3)	0
	Do not know	0	0	0
Knowledge of fluoride used in caries prevention	Yes	210 (81.3)	108 (78.2)	102 (85)
	No	48 (18.6)	30 (21.7)	18 (15)
Regular visits to the dentist can prevent dental problems	Agree	228 (88.3)	120 (86.9)	108 (90)
	Disagree	30 (11.6)	18 (13)	12 (10)
	Do not know	0	0	0

**Table 3:** Patients’ attitudes towards oral health.

Parameter	Evaluation Index	Total [*n* (%)]	Men [*n* (%)]	Women [*n* (%)]
The importance given to oral hygiene	Very important	180 (69.7)	84 (60.8)	96 (80)
	Important	72 (27.9)	48 (34.7)	24 (20)
	Less important	0	0	0
	Unimportant	6 (2.3)	6 (4.3)	0
The reason for avoiding the dentist	Fear of pain	54 (20.9)	36 (26)	18 (15)
	Distance	24 (9.3)	6 (4.3)	18 (15)
	It is not a priority	18 (6.9)	12 (8.6)	6 (5)
	High cost	102 (39.5)	42 (30.4)	60 (50)
	I do not hesitate	60 (23.2)	42 (30.4)	18 (15)
Pain is the main reason for visiting the dentist	Agree	198 (76.7)	120 (86.9)	78 (65)
	Disagree	60 (23.2)	18 (13)	42 (35)
	Do not know	0	0	0
Frequency of discomfort caused by dental problems in the last year	Often	162 (62.7)	90 (65.2)	72 (60)
	Occasional	24 (9.3)	12 (8.6)	12 (10)
	Rare	24 (9.3)	18 (13)	6 (5)
	Never	48 (18.6)	18 (13)	30 (25)
	Do not know	0	0	0

**Table 4:** Patients’ practices related to oral health.

Parameter	Evaluation Index	Total [*n*(%)]	Men [*n*(%)]	Women [*n*(%)]
Frequency of toothbrushing	Once a day	102 (39.5)	66 (47.8)	36 (30)
	Twice a day	120 (46.5)	60 (43.4)	60 (50)
	More than twice a day	24 (9.3)	6 (4.3)	18 (15)
	Less than once a day	12 (4.6)	6 (4.3)	6 (5)
Duration of toothbrushing	Less than 3 min	42 (16.2)	24 (17.3)	18 (15)
	3 min	72 (27.9)	48 (34.7)	24 (20)
	More than 3 min	126 (48.8)	54 (39.13)	72 (60)
	Do not know	18 (6.9)	12 (8.6)	6 (5)
Use of additional oral cleansing aids	Mouthwash	108 (41.8)	48 (34.7)	60 (50)
	Dental floss	72 (27.9)	36 (13.9)	36 (30)
	Tooth shower	0	0	0
	Do not use	78 (30.2)	54 (39.1)	24 (20)

**Table 5:** Eating habits.

**Parameter**	**Evaluation Index**	**Total [*n*(%)]**	**Men [*n*(%)]**	**Women [*n*(%)]**
Frequency of consumption of sweetened drinks	Never	0	0	0
	Once a week	18 (6.9)	12 (8.6)	6 (5)
	2–3 times/week	114 (44.1)	60 (43.4)	54 (45)
	Daily	126 (48.8)	66 (47.8)	60 (50)
Frequency of consumption of sweetened foods	Never	0	0	0
	Less than once a day	18 (6.9)	6 (4.3)	12 (10)
	Once a day	108 (41.8)	54 (39.1)	54 (45)
	More than once a day	132 (51.1)	78 (56.5)	54 (45)

## Data Availability

Not applicable.
